# Feasibility of single-incision distal gastrectomy for advanced gastric cancer

**DOI:** 10.1007/s00423-026-04033-2

**Published:** 2026-04-07

**Authors:** Jin Sil Jung, So Hyun Kang, Dongjae Jeon, Mira Yoo, Eunju Lee, Duyeong Hwang, Young Suk Park, Sang-Hoon Ahn, Yun-Suhk Suh, Hyung-Ho Kim

**Affiliations:** 1 Department of Surgery, Seoul National University Bundang Hospital, Seongnam, Korea; 2 Department of Surgery, Chung-Ang University Gwangmyeong Hospital, Gwangmyeong, Korea; 3 Department of Surgery, Samsung Medical Center, Seoul, Korea; 4 Department of Surgery, Dongguk University Ilsan Hospital, Goyang, Korea; 5 Department of Surgery, Seoul National University College of Medicine, Seoul, Korea; 6 Department of Surgery, School of Medicine, Sungkyunkwan University, Suwon, Korea; 7 Department of Surgery, Chung-Ang University College of Medicine, Seoul, Korea

**Keywords:** Laparoscopic surgery, Gastrectomy, Stomach neoplasm

## Abstract

**Background:**

While single-incision distal gastrectomy (SIDG) has been reported to be a potentially safe and feasible procedure for early gastric cancer (EGC), there is currently no study demonstrating the feasibility of SIDG for advanced gastric cancer (AGC) alone.

**Methods:**

Database of patients clinically diagnosed as AGC who underwent SIDG from November 2017 to September 2023 was retrospectively analyzed. Those who had distant metastasis, and those who underwent palliative surgery were excluded. Patient demographics, operation data, and postoperative complications were reviewed.

**Results:**

A total of 116 patients were included for the final analysis. Among them, 75 (64.7%) were male, and mean age was 62.7 ± 10.9 years. Average body mass index was 23.7 ± 3.3 kg/m2. The number of patients with stage pT2, pT3, and pT4a after surgery was 26 (22.4%), 31 (26.7%) and 23 (19.8%) respectively.

Mean operation time was 158.3 ± 56.8 min. and average estimated blood loss was 28.4 ± 60.4 ml. Average number of retrieved lymph nodes was 59.9 ± 21.6. Patients started their first soft fluid diet on an average of 2.3 ± 0.9 postoperative days, and first flatus was observed after 3.1 ± 0.9 postoperative days. Overall mean hospital stay was 7.5 ± 3.5 days. Early complications (within 30 days) greater than Clavien-Dindo grade III were found in 7 patients (6.0%) and late complications (after 30 days) were found in 4 patients (4.0%). No recurrence was observed during the follow-up period.

**Conclusion:**

Single-incision distal gastrectomy appears to be technically feasible and safe in carefully selected patients with clinically diagnosed advanced gastric cancer. However, further comparative studies with long-term follow-up are necessary to confirm its oncologic validity.

## Introduction

Gastric cancer is a common malignancy that is responsible for a significant number of cancer-related deaths worldwide [1]. Surgical resection remains the mainstay of treatment for localized gastric cancer, with curative resection resulting in the best long-term survival rates. With the results of Randomized Controlled Trials (RCT) such as the KLASS-01 [2], Laparoscopic Distal Gastrectomy (LDG) is now a standard treatment option for early gastric cancer. In addition to these results, RCTs of LDG for Advanced Gastric Cancer (AGC) such as the CLASS-01, KLASS-02, and the JLSSG0901 have been reported [3–5]. Results of these new trials show that lap. Distal Gastrectomy (DG) can also be a safe and feasible option for patients with AGC [6]. However, the optimal surgical approach for AGC still remains a matter of debate, with various techniques having been proposed over the years.

Single-incision laparoscopic surgery (SILS) is a surgical technique that has gained popularity in recent years due to its potential advantages, including improved cosmetic outcomes and reduced postoperative pain [7]. Single-incision distal gastrectomy (SIDG) is a specific application of SILS for the treatment of gastric cancer, which involves the removal of the distal portion of the stomach through a single incision in the umbilicus. SIDG has been shown to be a safe and feasible procedure for Early Gastric Cancer (EGC), with comparable surgical outcomes to conventional LDG [8, 9].

However, the feasibility of SIDG for AGC remains unclear. To our knowledge, no study has yet demonstrated the safety and feasibility of SIDG for AGC alone. Therefore, the aim of this study was to evaluate the feasibility of SIDG for AGC by analyzing a database of patients clinically diagnosed with AGC who underwent SIDG between November 2017 and September 2023. The results of this study will help to inform the selection of surgical approaches for patients with AGC and contribute to the ongoing debate surrounding the optimal surgical management of gastric cancer.

## Methods

This study was a retrospective analysis of a prospectively maintained database of patients who underwent SIDG for AGC between November 2017 and September 2023 at a single institution. Patients who were clinically diagnosed with AGC through endoscopy and radiologic imaging scans, and underwent SIDG were included in the study. Patients with distant metastasis, and those who underwent palliative surgery were excluded. This study was approved by the institutional review board (B-1707-409-007), and all patients provided written informed consent for the use of their medical data for research purposes.

Intraoperative and postoperative care of SIDG was performed according to methods previously described in the literature. Patients were prepared in the lithotomy position with the surgeon sitting between the legs, facing cephalad. A transumbilical incision of 2.5 cm was made [10]. To compensate for the lack of assistants in SIDG, a needlescopic device was sometimes used to assist in traction of tissues in some of the early cases of SIDG. In recent cases, intra-abdominal organ retractors and articulating laparoscopic instruments such as the Artisential (Livsmed) were used to facilitate the procedure.

For the analysis, patient demographics including age, sex, and body mass index (BMI) were recorded. The following surgical data were collected: operation time, estimated blood loss (EBL), and number of retrieved lymph nodes. Postoperative recovery parameters, including time to first soft fluid diet, time to first flatus, and length of hospital stay, were also included. Finally, the incidence of early (within 30 days) and late (after 30 days) postoperative complications was analyzed.

Statistical analyses were descriptive in nature. Continuous variables were expressed as mean ± standard deviation (SD), and categorical variables were expressed as frequencies and percentages. Overall survival was estimated using the Kaplan–Meier method. All statistical analyses were performed using R 3.6.1 (R Core Team, 2019).

## Results

**Fig. 1:**
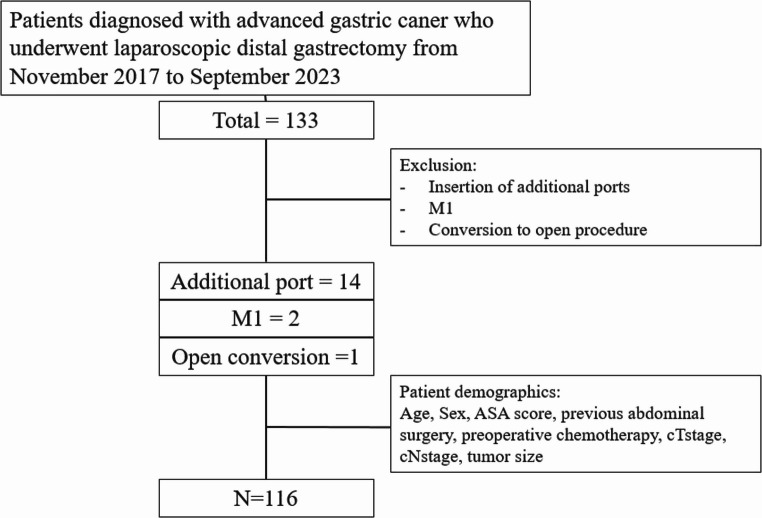
Flow diagram of patient selection for this study

A total of 133 patients diagnosed with advanced gastric cancer underwent laparoscopic distal gastrectomy from November 2017 to September 2023 (Fig. 1). Of these, 14 patients were excluded due to insertion of additional ports, 2 patients were excluded due to distant metastasis, and 1 patient was excluded due to conversion to an open procedure. Finally, data from a total of 116 patients who underwent SIDG for AGC were included in the final analysis.

The mean age was 62.7 ± 10.9 years, and the average BMI was 23.7 ± 3.3 kg/m2. The majority of the patients were male (64.7%, *n* = 75), while 35.3% (*n* = 41) were female. Reconstruction types following SIDG were predominantly Roux-en-Y (40.5%, *n* = 47), followed by Billroth I (32.8%, *n* = 38), Billroth II (21.6%, *n* = 25), and Uncut Roux-en-Y (5.2%, *n* = 6). The lymph node dissection primarily consisted of D2 dissection (92.2%, *n* = 107), followed by D2 + dissection (6.9%, *n* = 8), and D1 + dissection (0.9%, *n* = 1). The mean tumor size was 4.4 ± 2.1 cm. Regarding the T stage, 6.9% (*n* = 8) of patients were classified as T1a, 24.1% (*n* = 28) as T1b, 22.4% (*n* = 26) as T2, 26.7% (*n* = 31) as T3 and 19.8% (*n* = 23) as T4a. The distribution of N stage was as follows: N0 in 41.4% (*n* = 48) of patients, N1 in 21.6% (*n* = 25), N2 in 19.8% (*n* = 23), N3a in 13.8% (*n* = 16), and N3b in 3.4% (*n* = 4). Other patient characteristics are summarized in Table 1.

**Table 1:** Patient demographics and pathologic outcomes

Group	*N* = 116
Age (years)	62.7 ± 10.9
Sex	
Male	75 (64.7%)
Female	41 (35.3%)
Body mass index (kg/m2)	23.7 ± 3.3
ASA score*	
1	33 (28.4%)
2	66 (56.9%)
3	16 (13.8%)
4	1 (0.9%)
History of previous abdominal operation	30 (25.9%)
Reconstruction type	
Billroth I	38 (32.8%)
Billroth II	25 (21.6%)
Roux-en-Y	47 (40.5%)
Uncut Roux-en-Y	6 (5.2%)
Lymph node dissection	
D1+	1 (0.9%)
D2	107 (92.2%)
D2+	8 (6.9%)
Lymphatic invasion	71 (61.7%)
Vascular invasion	14 (12.2%)
Perineural invasion	51 (44.3%)
Other organ invasion	0 (0%)
Tumor size (cm)	4.4 ± 2.1
Proximal margin (cm)	3.4 ± 2.1
Distal margin (cm)	4.6 ± 2.8
T stage	
T1a	8(6.9%)
T1b	28 (24.1%)
T2	26 (22.4%)
T3	31 (26.7%)
T4a	23 (19.8%)
N stage	
N0	48 (41.4%)
N1	25 (21.6%)
N2	23 (19.8%)
N3a	16 (13.8%)
N3b	4 (3.4%)

*American Society of Anesthesiologists

The mean operation time was 158.3 ± 56.8 min, and the average EBL was 28.4 ± 60.4 ml (Table 2). The mean proximal margin was 3.4 ± 2.1 cm, while the mean distal margin was 4.6 ± 2.8 cm. The average number of retrieved lymph nodes was 59.9 ± 21.6, and the mean number of positive nodes was 3.9 ± 11.1. Patients started their first soft fluid diet on average 2.3 ± 0.9 days after surgery, and the first flatus was observed after an average of 3.1 ± 0.9 postoperative days. The overall mean hospital stay was 7.5 ± 3.5 days.

**Table 2:** Operative outcomes

Group	*N* = 116
Operation time (minutes)	158.3 ± 56.8
Estimated blood loss (ml)	28.4 ± 60.4
Hospital stay (days)	7.5 ± 3.5
Time to soft fluid diet (days)	2.3 ± 0.9
Time to first flatus (days)	3.1 ± 0.9
Number of retrieved lymph nodes	59.9 ± 21.6
Number of positive lymph nodes	3.9 ± 11.1

In terms of postoperative complications (Table 3), 19.0% (*n* = 22) of patients experienced early complications (within 30 postoperative days) including Clavien-Dindo Grade I. Among the early complications, 15.5% (*n* = 18) had complications of Clavien-Dindo Grade II or higher, and 6.0% (*n* = 7) had complications of Clavien-Dindo Grade III or higher. The 7 patients with Clavien-Dindo grade III or higher consisted of wound morbidity (*n* = 1), motility disorder (*n* = 1), intra-abdominal abscess (*n* = 4) and internal hernia (*n* = 1, Petersen’s hernia). Late complications (more than 30 postoperative days) were observed in 3.4% (*n* = 4) of patients which consisted of anastomosis stricture (*n* = 1), incisional hernia (*n* = 1), internal hernia (*n* = 1) and delayed gastric emptying (*n* = 1). No perioperative deaths occurred within 30 or 90 days after surgery.

**Table 3:** Postoperative complications

Group	*N* = 116
Early complications	22 (19.0%)
Early complications ≥ C-D^a^ grade II	18 (15.5%)
Early complications ≥ C-D^a^ grade IIIa	7 (6.0%)
Wound morbidity	1
Motility disorder	1
Intra-abdominal abscess	4
Other early complication (Peterson’s hernia)	1
Late complications	4 (3.4%)
Anastomosis stricture	1
Incisional hernia	1
Internal hernia	1
Delayed gastric emptying	1
Perioperative mortality	0 (0.0%)
Median follow-up duration (months)	44.4

^a^Clavien-Dindo grade

**Fig. 2:**
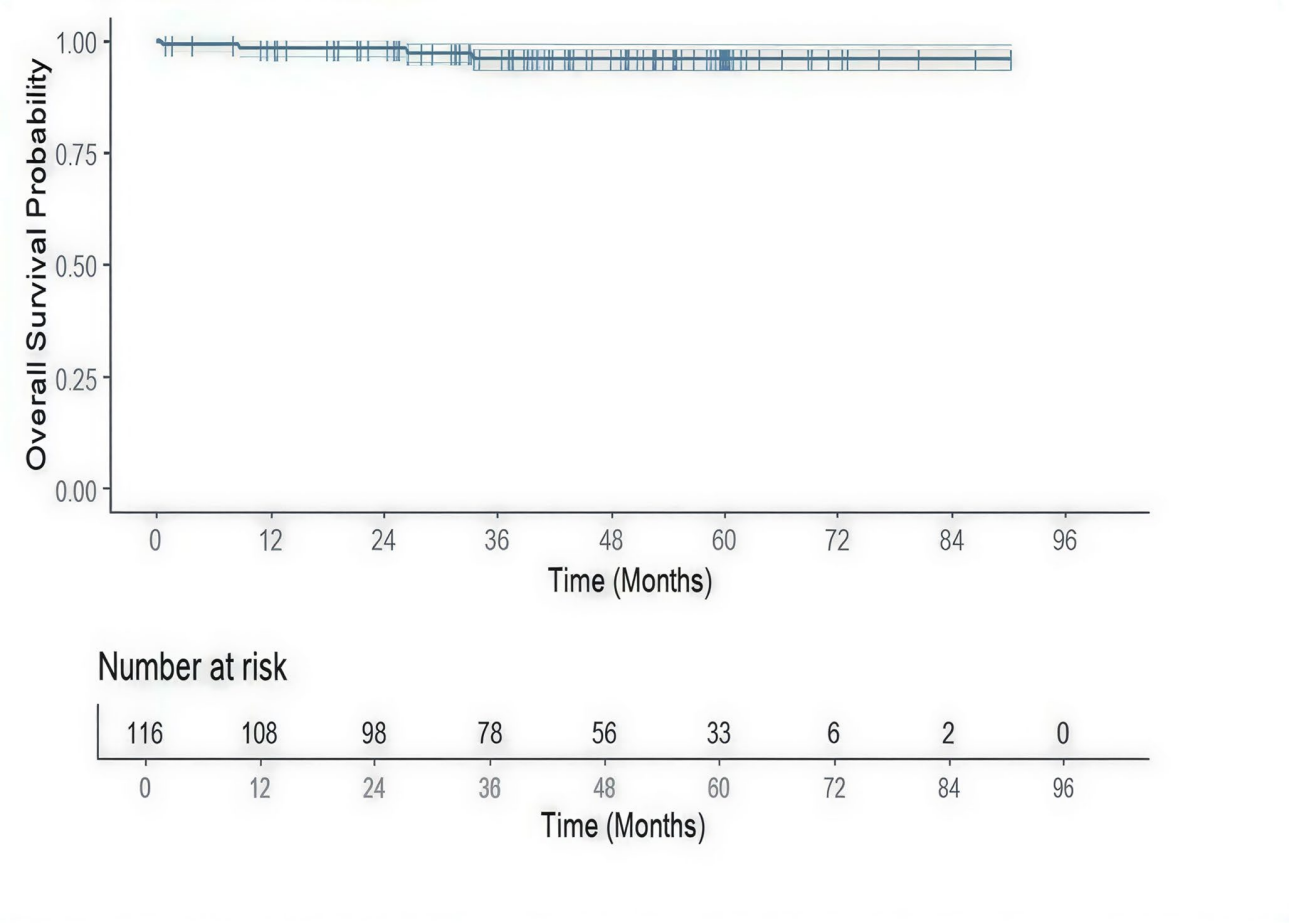
Kaplan-Meier curve showing overall survival of patients with clinically diagnosed advanced gastric cancer who underwent single-incision distal gastrectomy

No recurrence was observed during the follow-up period. At a median follow-up duration of 44.4 months, the Kaplan-Meier estimated overall survival (OS) rate was 96.0%. Four deaths occurred during the follow-up period and were included in the overall survival analysis. The survival probability remained consistently high throughout the entire follow-up period, with minimal decline over time. (Fig. 2)

## Discussion

Several studies have reported the potential benefits of single-incision surgery, such as reduced postoperative pain, improved cosmetic outcomes, and shorter hospital stays [11]. These advantages have been particularly evident in early gastric cancer (EGC), where single-incision laparoscopic surgery (SILS) has demonstrated comparable oncologic outcomes to conventional laparoscopic gastrectomy [2, 12, 13]. However, the feasibility and safety of single-incision distal gastrectomy (SIDG) for advanced gastric cancer (AGC) remain less well established, and the current evidence is limited.

SIDG for AGC is a technically demanding procedure due to the complexity of the disease and the need to maintain strict oncologic principles. One of the main challenges is the limited range of motion and reduced instrument triangulation, which can make dissection and reconstruction more difficult [14–16]. Additionally, the procedure may require longer operative times because of the meticulous dissection needed to ensure clear margins and adequate lymphadenectomy [15]. Despite these challenges, with increasing experience and the development of new surgical devices and techniques, the difficulty of SIDG for AGC can be overcome.

Our study aimed to address this gap in the literature by providing preliminary data on the feasibility of SIDG for AGC. The results suggest that, despite the technical demands, SIDG can be performed with acceptable operative parameters. The mean operation time was approximately 158 min, which is comparable to other minimally invasive approaches for AGC. The average number of retrieved lymph nodes was over 59, indicating that oncologic principles were maintained. Postoperative recovery was favorable, with patients resuming soft fluid intake around 2.3 days and passing flatus by day 3 on average. The overall hospital stay was about 7.5 days, and the complication rate was relatively low, with only 19% experiencing early postoperative issues—most of which were manageable. Importantly, during the follow-up period, no recurrences were observed, and the median overall survival rate was high at 96% over 2.5 years, supporting the mid-term oncologic safety of SIDG in carefully selected patients.

Current data on SIDG for AGC are limited but suggest outcomes comparable to laparoscopic distal gastrectomy (LDG). For example, Omori et al. [13] reported in a meta-analysis that surgery time was shorter in the single-incision group (162 vs. 182 min, *p* = 0.0399) for EGC, despite the increased complexity of AGC. Our mean operative time of 158.3 min aligns with these findings, especially considering that our procedures were mostly performed by experienced surgeons at a teaching hospital, and that the single incision reduces wound closure time. Furthermore, a meta-analysis comparing LDG to open distal gastrectomy (ODG) in AGC showed that LDG had fewer postoperative complications and shorter hospital stays than ODG [17]. In our cohort, the number of retrieved lymph nodes was significantly higher (59.3 ± 22.1) compared to other studies, which is crucial for accurate staging and prognosis. Additionally, the early complication rate in our study (19%) was slightly lower than in some reports, and comparable to rates seen in MLDG in EGC [18]. In the KLASS-02 trial [4], a multicenter randomized controlled study of clinically diagnosed AGC patients, a 30-day morbidity rate of 16.6% and a 90-day mortality rate of 0.4% was reported after laparoscopic distal gastrectomy for AGC. This suggests that SIDG might be a feasible option in some selected patients with AGC.

Given the technical complexity of SIDG, understanding the learning curve is crucial. Kang et al. [15] reported that an experienced surgeon requires fewer than 20 cases to stabilize operative times. However, specific analyses of the learning curve for SIDG in AGC are still lacking and should be addressed in future studies. To facilitate SIDG in challenging cases, several new surgical devices and techniques have been developed [19, 20]. The use of flexible endoscopes improves visualization and reduces instrument clashing; in this study, a 10-mm HD flexible scope or a 3D flexible scope (Olympus, Tokyo, Japan) was employed and fixed to a scope holder [15]. Articulating laparoscopic devices such as ArtiSential^®^ (LivsMed) enable fine dissection in reduced-port settings [21–24]. Moreover, advancements in robotic platforms like Da Vinci have provided improved dexterity and range of motion, which may be instrumental in the future of SILS techniques [13, 14, 25, 26].

This study has several important limitations that should be acknowledged. First, the present study was designed as a single-arm cohort without a comparator group such as multiport laparoscopic or open distal gastrectomy. Therefore, no direct conclusions regarding the comparative oncologic superiority or non-inferiority of SIDG can be drawn. Rather, the primary aim of this study was to evaluate the early technical feasibility and perioperative safety of SIDG in patients clinically diagnosed with AGC. Second, although the study focused on patients with clinically diagnosed AGC, pathological examination revealed that a proportion of patients had pT1 disease. This discrepancy reflects the prospective enrollment strategy based on preoperative clinical staging using computed tomography and endoscopic ultrasonography, which is known to have inherent limitations in accurately predicting pathological T stage. Similar stage migration has been reported in other prospective studies of AGC, and therefore inclusion of pT1 cases was unavoidable in a clinically staged cohort. Third, although the median follow-up duration of 44.4 months allowed assessment of mid-term outcomes and included evaluation of 3-year disease-free survival for the majority of patients, this duration remains insufficient to draw definitive conclusions regarding long-term oncologic outcomes for AGC, particularly in the absence of a control group. While previous studies have suggested that 3-year disease-free survival may reasonably predict 5-year overall survival in gastric cancer, longer follow-up and comparative studies are required to further validate the oncologic adequacy of SIDG for AGC. In addition, it should also be noted that recurrence was not a primary endpoint of this study, and systematic assessment of long-term oncologic outcomes was beyond the original scope of the study design. Therefore, the absence of observed recurrence should be interpreted with caution and does not imply definitive long-term oncologic safety. Fourth, the relatively small sample size of 116 patients may restrict the statistical power and generalizability of the results. Additionally, the study did not analyze the learning curve specific to SIDG in AGC, which is an important factor influencing surgical outcomes and surgeon proficiency. Future research with larger cohorts and extended follow-up is therefore necessary to validate these preliminary results and to better define the long-term safety and effectiveness of SIDG for AGC.

## Conclusion

In conclusion, this study demonstrates that single-incision distal gastrectomy is technically feasible and can be performed with acceptable perioperative outcomes in carefully selected patients with clinically diagnosed advanced gastric cancer. Further large-scale comparative studies with long-term follow-up are required to define the role of single-incision distal gastrectomy in the surgical management of advanced gastric cancer.

## Data Availability

No datasets were generated or analysed during the current study.

## References

[1] Bray F, Ferlay J, Soerjomataram I, Siegel RL, Torre LA, Jemal A (2018) Global cancer statistics 2018: GLOBOCAN estimates of incidence and mortality worldwide for 36 cancers in 185 countries. CA Cancer J Clin 68:394–424. 10.3322/caac.21492 30207593 10.3322/caac.21492

[2] Kim HH, Han SU, Kim MC, Kim W, Lee HJ, Ryu SW, Cho GS, Kim CY, Yang HK, Park DJ, Song KY, Lee SI, Ryu SY, Lee JH, Hyung WJ (2019) Effect of laparoscopic distal gastrectomy vs open distal gastrectomy on long-term survival among patients with stage I gastric cancer: The KLASS-01 randomized clinical trial. JAMA Oncol 5:506–513. 10.1001/jamaoncol.2018.6727 30730546 10.1001/jamaoncol.2018.6727 PMC6459124

[3] Huang C, Liu H, Hu Y, Sun Y, Su X, Cao H, Hu J, Wang K, Suo J, Tao K, He X, Wei H, Ying M, Hu W, Du X, Yu J, Zheng C, Liu F, Li Z, Zhao G, Zhang J, Chen P, Li G (2022) CLASS-01 Group. Laparoscopic vs open distal gastrectomy for locally advanced gastric cancer: Five-year outcomes from the CLASS-01 randomized clinical trial. JAMA Surg 157:9–17. 10.1001/jamasurg.2021.5104 34668963 10.1001/jamasurg.2021.5104 PMC8529527

[4] Hyung WJ, Yang HK, Park YK, Lee HJ, An JY, Kim W, Kim HI, Kim HH, Ryu SW, Hur H, Kim MC, Kong SH, Cho GS, Kim JJ, Park DJ, Ryu KW, Kim YW, Kim JW, Lee JH, Han SU (2020) Long-term outcomes of laparoscopic distal gastrectomy for locally advanced gastric cancer: The KLASS-02-RCT randomized clinical trial. J Clin Oncol 38:3304–3313. 10.1200/JCO.20.01210 32816629 10.1200/JCO.20.01210

[5] Etoh T, Ohyama T, Sakuramoto S, Tsuji T, Lee SW, Yoshida K, Koeda K, Hiki N, Kunisaki C, Tokunaga M, Otsubo D, Takagane A, Misawa K, Kinoshita T, Cho H, Doki Y, Nunobe S, Shiraishi N, Kitano S, JLSSG Group (2023) Five-year survival outcomes of laparoscopy-assisted vs open distal gastrectomy for advanced gastric cancer: The JLSSG0901 randomized clinical trial. JAMA Surg 158:445–454. 10.1001/jamasurg.2023.0096 36920382 10.1001/jamasurg.2023.0096 PMC10018406

[6] Japanese Gastric Cancer Association (2022) Japanese gastric cancer treatment guidelines 2021 (6th edition). Gastric Cancer 26:1–25. 10.1007/s10120-022-01331-8 36342574 10.1007/s10120-022-01331-8 PMC9813208

[7] Omori T, Yamamoto K, Hara H, Shinno N, Yamamoto M, Sugimura K, Wada H, Takahashi H, Yasui M, Miyata H, Ohue M, Yano M, Sakon M (2021) A randomized controlled trial of single-port versus multi-port laparoscopic distal gastrectomy for gastric cancer. Surg Endosc 35:4485–4493. 10.1007/s00464-020-07955-0 32886237 10.1007/s00464-020-07955-0

[8] Kang SH, Yoo M, Hwang D, Lee E, Lee S, Park YS, Ahn SH, Suh YS, Kim HH (2023) Postoperative pain and quality of life after single-incision distal gastrectomy versus multiport laparoscopic distal gastrectomy for early gastric cancer – a randomized controlled trial. Surg Endosc 37:2095–2103. 10.1007/s00464-022-09709-6 36307602 10.1007/s00464-022-09709-6 PMC9616415

[9] Kang SH, Lee E, Lee S, Park YS, Ahn SH, Park DJ, Kim HH (2022) Long-term outcomes of single-incision distal gastrectomy compared with conventional laparoscopic distal gastrectomy: A propensity score–matched analysis. J Am Coll Surg 234:340–351. 10.1097/XCS.0000000000000052 35213497 10.1097/XCS.0000000000000052

[10] Omori T, Oyama T, Akamatsu H, Tori M, Ueshima S, Nishida T (2011) Transumbilical single-incision laparoscopic distal gastrectomy for early gastric cancer. Surg Endosc 25:2400–2404. 10.1007/s00464-010-1563-3 21298524 10.1007/s00464-010-1563-3

[11] Kim YW, Baik Y, Yun YH, Nam BH, Kim DH, Choi I, Bae JM (2008) Improved quality of life outcomes after laparoscopy-assisted distal gastrectomy for early gastric cancer. Ann Surg 248:721–727. 10.1097/SLA.0b013e318185e62e 18948798 10.1097/SLA.0b013e318185e62e

[12] Lee JH, Yom CK, Han HS (2009) Comparison of long-term outcomes of laparoscopy-assisted and open distal gastrectomy for early gastric cancer. Surg Endosc 23:1759–1763. 10.1007/s00464-008-0198-0 19057958 10.1007/s00464-008-0198-0

[13] Omori T, Fujiwara Y, Moon J, Sugimura K, Miyata H, Masuzawa T, Kishi K, Miyoshi N, Tomokuni A, Akita H, Takahashi H, Kobayashi S, Yasui M, Ohue M, Yano M, Sakon M (2016) Comparison of single-incision and conventional multi-port laparoscopic distal gastrectomy with D2 lymph node dissection for gastric cancer: A propensity score–matched analysis. Ann Surg Oncol 23:817–824. 10.1245/s10434-016-5485-8 27510844 10.1245/s10434-016-5485-8

[14] Huang KH, Lan YT, Fang WL, Chen JH, Lo SS, Li A, Chiou SH, Wu CW, Shyr YM (2014) Comparison of the operative outcomes and learning curves between laparoscopic and robotic gastrectomy for gastric cancer. PLoS ONE 9:e111499. 10.1371/journal.pone.0111499 25360767 10.1371/journal.pone.0111499 PMC4216064

[15] Kang SH, Cho YS, Min SH, Park YS, Ahn SH, Park D, Kim HH (2019) Early experience and learning curve of solo single-incision distal gastrectomy for gastric cancer: A review of consecutive 100 cases. Surg Endosc 33:3412–3418. 10.1007/s00464-018-06638-1 30604257 10.1007/s00464-018-06638-1

[16] Lee B, Lee Y, Park YS, Ahn SH, Park D, Kim HH (2018) Learning curve of pure single-port laparoscopic distal gastrectomy for gastric cancer. J Gastric Cancer 18:182–188. 10.5230/jgc.2018.18.e20 29984068 10.5230/jgc.2018.18.e20 PMC6026715

[17] Yan Y, Ou C, Cao S, Hua Y, Sha Y (2023) Laparoscopic vs. open distal gastrectomy for locally advanced gastric cancer: A systematic review and meta-analysis of randomized controlled trials. Front Surg 10:1127854. 10.3389/fsurg.2023.1127854 36874456 10.3389/fsurg.2023.1127854 PMC9982133

[18] Lee B, Youn SI, Lee K, Won Y, Min S, Lee YT, Park YS, Ahn SH, Park DJ, Kim HH (2021) Comparing the short-term outcomes and cost between solo single-incision distal gastrectomy and conventional multiport totally laparoscopic distal gastrectomy for early gastric cancer: A propensity score-matched analysis. Ann Surg Treat Res 100:67–75. 10.4174/astr.2021.100.2.67 33585351 10.4174/astr.2021.100.2.67 PMC7870426

[19] Suh YS, Lee HJ, Yang HK (2016) Single incision gastrectomy for gastric cancer. Transl Gastroenterol Hepatol 1:41. Available from: https://tgh.amegroups.org/article/view/973/html. Accessed 24 Aug 2025 10.21037/tgh.2016.05.05 PMC5244807 28138608

[20] Suh YS, Park JH, Kim TH, Huh YJ, Son YG, Yang JY, Kong SH, Lee HJ, Yang HK (2015) Unaided stapling technique for pure single-incision distal gastrectomy in early gastric cancer: Unaided delta-shaped anastomosis and uncut Roux-en-Y anastomosis. J Gastric Cancer 15:105–112. 10.5230/jgc.2015.15.2.105 26161283 10.5230/jgc.2015.15.2.105 PMC4496436

[21] Hong SK, Shin E, Lee KW, Yoon KC, Lee JM, Cho JH, Yi NJ, Suh KS (2019) Pure laparoscopic donor right hepatectomy: Perspectives in manipulating a flexible scope. Surg Endosc 33:1667–1673. 10.1007/s00464-018-6594-1 30465077 10.1007/s00464-018-6594-1

[22] Kim A, Lee CM, Park S (2021) Is it beneficial to utilize an articulating instrument in single-port laparoscopic gastrectomy? J Gastric Cancer 21:38–48. 10.5230/jgc.2021.21.e2 33854812 10.5230/jgc.2021.21.e2 PMC8020002

[23] Lee E, Lee K, Kang SH, Lee S, Won Y, Park YS, Ahn SH, Suh YS, Kim HH (2021) Usefulness of articulating laparoscopic instruments during laparoscopic gastrectomy for gastric adenocarcinoma. J Minim Invasive Surg 24:35–42. 10.7602/jmis.2021.24.1.35 35601278 10.7602/jmis.2021.24.1.35 PMC8966001

[24] Jin HY, Ibahim AM, Bae JH, Lee CS, Han SR, Lee IK, Lee DS, Lee YS (2022) Initial experience of laparoscopic complete mesocolic excision with D3 lymph node dissection for right colon cancer using Artisential^®^, a new laparoscopic articulating instrument. J Minim Access Surg 18:235–240. 10.4103/jmas.JMAS_88_21 35313433 10.4103/jmas.JMAS_88_21 PMC8973474

[25] Loureiro P, Barbosa JP, Vale JF, Barbosa J (2023) Laparoscopic versus robotic gastric cancer surgery: Short-term outcomes—systematic review and meta-analysis of 25,521 patients. J Laparoendosc Adv Surg Tech A 33:782–800. 10.1089/lap.2023.0136 37204324 10.1089/lap.2023.0136

[26] Darwich I, Abuassi M, Aliyev R, Scheidt M, Alkadri MA, Hees A, Demirel-Darwich S, Chand M, Willeke F (2022) Early experience with the ARTISENTIAL^®^ articulated instruments in laparoscopic low anterior resection with TME. Tech Coloproctol 26:373–386. 10.1007/s10151-022-02588-y 35141794 10.1007/s10151-022-02588-y PMC9018813

